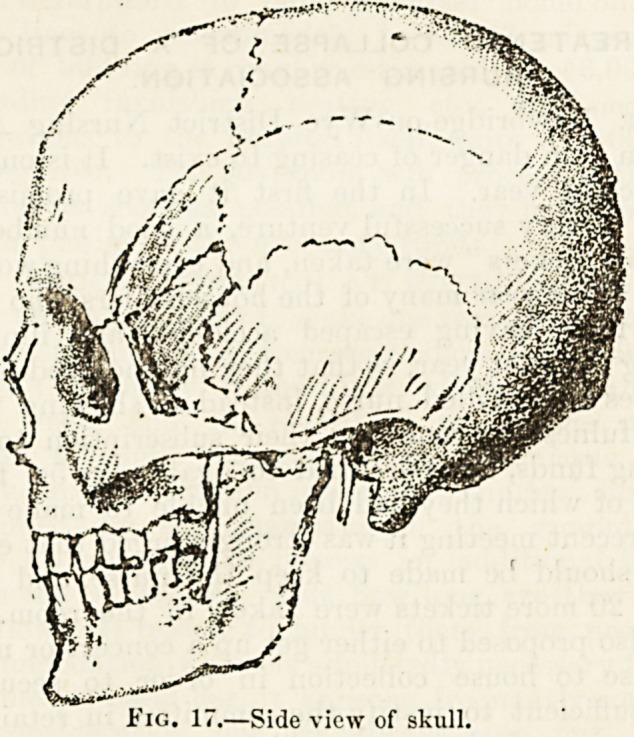# The Hospital. Nursing Section

**Published:** 1901-12-28

**Authors:** 


### The Hospital.

### Nursing Section.

Contributions for this Section of "The Hospital" should be addressed to the Editor, "The Hospital" Nursing Section, 28 & 29 Southampton Street, Strand, London, W.C.

No. 796.—Vol. XXXI. *SATURDAY, DECEMBER* 28, 1901.

### Notes on News from the Nursing World.

### A TOUCHING STORY OF QUEEN VICTORIA.

At the opening meeting of the Gordon League, Mr. Harold Boulton, who narrated the history of the establishment and progress of the Queen Victoria Jubilee Institute for Nurses, said that the late Queen a very short while before her death visited a military hospital and asked one man who had been terribly mutilated in a South African battle if there was anything she could do for him. "Only to thank the nurse," was the soldier's faint answer, and the Queen gravely laid her hand on the Victoria nurse's shoulder and said, "I thank you, my daughter, for your goodness to my son."

### THE NURSES FOR THE CONCENTRATION CAMPS.

In addition to the two ladies whose names we mentioned last week, the following have been appointed by the Colonial Secretary to nurse in the Concentration Camps in South Africa :—Matrons : Misses Halkett, Little, Stevens, and Mackenzie. Nurses : Mrs. Deas, Misses Gordon, Shelley, Underwood, Wilkie, Stevens, J. A. Fraser, Henry, Garvie, Lord, Craig, Hunter, List, Carter, J. Fraser, Hobson, and Mallet. Some of these ladies sailed in the *Plassy* on the 16th, and the remainder go by the *Simla*. Miss Wilkie is the former matron of the Halifax Workhouse Hospital, and Miss Gordon is one of the nurses who resigned at the same time. Miss Shelley, who was trained at the General Hospital, Birmingham, has already served in South Africa.

### THE QUESTIONS OF THE COLONIAL SECRETARY.

Each applicant for a post as nurse in the Concentration Camps has to refer the Secretary of State for the Colonies to some one well acquainted with her, who in her turn is required to reply to a number of searching questions. They run as follows :—(1) Will you be good enough to state how long you have been acquainted with the applicant ? (2) From what circumstances does your knowledge of her arise ? (3) While you were acquainted with her was she honest, sober, and generally well-conducted ? (4) Was her health good ? (5) If she has been employed under you, will you state the nature of her duties and how she discharged them ? (6) Will you state the cause of her leaving, whether by voluntary resignation, dismissal, or otherwise ? (7) Do you consider her to be professionally qualified to undertake the duties of such an appointment as she now desires ? (8) Are you aware of any circumstances tending to disqualify her for such an appointment ? The Secretary of State adds that he will be glad to receive any information which the referee may have to give respecting the character or qualifications of the applicant further than is contained in the answers to his questions.

### THE FOOD QUESTION.

The "Nurses' Never," which has attracted so much attention, and the letters commenting upon it, have not been published in The Hospital in order to provoke "immense amusement." Nor do we at all admire the tone of some of our correspondents' contributions. There has, we think, been more to excite serious reflection than merriment in the controversy. "A Sister" in to-day's issue deals, if somewhat ruthlessly, with a point which must have occurred to many of our readers. It is not, as a rule, the young woman who has left a comfortable home and been used to many refinements that complains of her food at the hospital, but the young woman who has taken up nursing with the idea of "bettering" herself. We do not assert that the diet of night nurses is always what it should be. "N. G." showed last week that complaints may sometimes be completely justified. Moreover, we concur in the opinion that night nurses merit at least as much consideration as day nurses in respect to the variety and the quality of their food. The matron of the Cancer Hospital, who elsewhere gives details of the diet of her night nurses, only confirms our conviction that so far as most hospitals and many Poor Law infirmaries are concerned, the diet for both day and night nurses is as liberal, as varied, and as good, as can reasonably be expected ; and we are afraid that some nurses while not living up to the standard of the "Nurses' Never," because it is beyond them, live down to a standard that begins and ends with their own personal needs. We hope that they are only a small minority.

### THE ROYAL FREE HOSPITAL.

Quite a week before Christmas, a pile of evergreens in the courtyard of the Royal Free Hospital, Gray's Inn Road, and a collection of Chinese lanterns seen through a window betokened Christmas ; the first belonged to the hospital for use in decoration, and the second was the property of the students. On Tuesday and Thursday in the week preceding Christmas, carols were sung in the wards by the students, and the effect, with lights turned low, a blazing fire and large lanterns carried on poles with holly boughs, was very pretty. The students, in the varied hues of evening dress, were accompanied by the house surgeon, who played the piano ; and the familiar "Noel, Noel," and "Hark ! the Herald Angels" sounded very sweet across the gloom of the courtyard as one approached. After the singing, a great knocking at the door was followed by the entrance of Father Christmas, in regulation scarlet and cotton-wool, and "Sister" was requested to conduct him to each bed, where bundles of clothing were deposited ; he made his exit to the sound of popping crackers, to repeat the performance in the next ward, and subsequently the performers were entertained by the sisters.

### THE THANKS OF THE NORWEGIAN GOVERNMENT.

In connection with the loss of the *Erato* last month the Norwegian Government, through the medium of the Vice-Consul for Sweden and Norway at Middlesbrough, have expressed their thanks to all who rendered help on the occasion of the wreck. Special reference is made to the valuable assistance rendered by the matron and nursing staff of the Miners' Hospital at Skinningrove. The Vice-Consul in his letter says :—

> By the indefatigable efforts of the hospital matron, Miss Edith A. Weale, and other willing helpers, Axelsen was, after some time, resuscitated, and removed to the Miners' Hospital, where he was found to have been seriously injured on the head by the wreckage. The careful and excellent nursing of Miss Weale and her staff during these three weeks have gradually restored the boy, and I am glad to say he is now so far recovered that I have been able to send him home. But, not content with nursing him back to life, Miss Weale in generous sympathy with him and his poor mother, who is a widow, has, from a number of her friends, raised a subscription for him amounting to £6 12s. 2½d., which will enable him to purchase a new outfit when he is strong enough to resume the duties of his calling.

It is gratifying to find the services of English nurses thus handsomely recognised by a foreign Government.

### SICK-ROOM COOKERY.

The nurses of three Bradford hospitals are shortly going to receive lessons in sick-room cookery from Miss Maude Mason, of Leeds, and early in the new year she will begin a course of lectures to members of the Ambulance Guild at Dewsbury, between 60 and 70 in number. In our issue of next week we shall publish the first of a short series of articles by Miss Mason.

### FORMATION OF A CLUB AT GLASGOW.

Last week there was opened in Glasgow a nurses' guild and club. The committee have secured a flat at Charing Cross, a central part of the City. The rooms are very daintily and artistically furnished with antique furniture ; the soft colouring of carpets and wall-papers is especially restful. They comprise sitting- and writing-room, supplied with newspapers and magazines ; and a bedroom for nurses who may wish to stay a few days. The rooms on the opening day were bright with flowers and plants, and an "at home" was held, which was attended by medical men, clergymen, and others interested, as well as by nurses from the various infirmaries. It is hoped that, after the trouble the committee have taken in starting the club, nurses will do their part in keeping it up.

### CURIOUS CHARGES AGAINST IRISH NURSES.

Unusual allegations against two nurses at Kanturk Workhouse Hospital, county Cork, have been the subject of inquiry by Dr. E. C. Bigger, a medical inspector under the Irish Local Government Board. Until the report of Dr. Bigger has been considered by the Board, and the decision of the latter made known, comment would, of course, be improper. But it may be stated that the nurses are accused by certain male patients in the hospital of skimming the cream off the milk of the patients to use for their own tea and to make oaten meal cakes. It was also asserted that the nurses did not change the blankets as often as they should, and that the poultices were not put on in accordance with the doctor's instructions. To these charges an emphatic denial is given, and the medical officer and surgeon both declared that they always found the nurses discharge their duties in an excellent and capable manner. Dr. Bigger, at the close of the inquiry, asked the medical officer if, having regard to the criticisms they had heard, and all the circumstances disclosed, he was satisfied that he had a sufficient nursing staff. The answer was that in the event of a vacancy the appointment of a trained matron would be desirable, and Dr. Bigger himself then suggested that in any case a trained nurse who would act as superintendent should be appointed. It is, therefore, highly probable that whatever may be the conclusion of the Irish Local Government Board in reference to the charges against the nurses, the investigation will be fraught with satisfactory results.

### A NURSES' HOME FOR THE GLOUCESTER HOSPITAL.

The General Infirmary at Gloucester and the Gloucestershire Eye Institution—as the county hospital in the Western city is somewhat cumbrously named—was founded a century and a half ago, but it is at least 30 years since any large structural additions were carried out. The governors have at length realised that the accommodation is now totally inadequate to the needs of a modern hospital, and particularly so in regard to the provision for the nursing staff. Thirty years ago eleven nurses staffed the same number of wards as are open to-day, which are now tended by 34 nurses, including probationers, with the occasional help in times of pressure of out-nurses. The consequence is that the institution is seriously overcrowded, and a committee of the governing body have decided that the arrangements of the ward annexes are contrary to the recognised principles of sanitation. It has therefore been determined to build a nurses' home on up-to-date lines to accommodate in all 43 nurses. The cost of building is roughly estimated at £6,000, but including furnishing, heating, etc., and necessary alterations to the hospital itself, quite £10,000 will be required. The governors will proceed with their scheme as soon as possible, and they have appealed to the public for the necessary funds.

### PROCRASTINATION AT ROTHERHITHE.

It seems necessary to make one remark in reference to the statements of a correspondent concerning St. Olave's Infirmary, Rotherhithe, which we published last week, and in regard to which the matron makes a slight correction in this issue. The delay in the creation of a new nurses' home is defended by the guardians on the ground that they do not like to impose an additional burden upon the ratepayers. But how long are they going to allow this reason to hinder them from providing accommodation which is admittedly necessary ? The scheme in hand has, it appears, frequently come up for consideration, but has always been put on one side. The nurses of St. Olave's Infirmary seem to be well treated and the staff to be adequate. But the indefinite postponement of the execution of a scheme which, we presume, was proposed because of urgent need and not for a joke, on the ground of saving the pockets of the ratepayers, savours of unwise procrastination.

### A TRIBUTE TO THE NURSES OF A LONDON HOSPITAL.

The driver of an omnibus going along Piccadilly in the direction of Hyde Park Corner the other day turned his head and thus "opened conversation" with a nurse who was a top front-seat passenger:—"You are a 'orspital nurse, Miss, I take it, from your dress?" "I am," she answered, and, knowing the ways of the "London 'busmen," she waited with a little amusement for more. "Ah, well, there's a-many a young woman took up with that work nowadays, and it's the most noblest work going, it is, 'specially if it is took up with aright." Silence for a minute. "It warn't long since I come out of a 'orspital myself. I am a reading man, I am, and I don't drink, nor yet smoke, and my money I takes it all home to the wife. Poor soul, she were cut up when I took ill, and the doctor he said I had better go into the 'orspital, as I should come round it quicker there." "And where did you go?" the nurse asked. "Why, to the 'London Temperance'; it was this yere pneumonia I had, and I was in five weeks." "Good to me?" he went on, being fairly "wound up" now and requiring no "prompting"—"they was all good; but there was two 'specially so. And that there young woman (nurse, I should say) that a-minded of me by night—if ever there was a hangel on earth it was her. There wasn't nothing too good for me, nor too much trouble to do for me; and me only a rough chap, as you see, Miss. Ay, it's a good thing there's such places to go to. It's a good 'orspital is the 'London Temperance;' and as long as me and my old missus are alive we shall be thankful for the day as I went in there. And them two young women—well, hangels on earth they was; there is no other word for them, and I shall never forget them—never, as long as I live." "Good day to you, Miss; good day," as the nurse got down at the "corner." "And I am proud to have met you on my 'bus; I am that."

### THREATENED COLLAPSE OF A DISTRICT NURSING ASSOCIATION.

The Newbridge-on-Wye District Nursing Association is in danger of ceasing to exist. It is only in its second year. In the first it gave promise of being a very successful venture, a good number of "family tickets" were taken, and everything worked well. This year many of the householders who were subscribers having escaped accidents and illnesses during the past year, so that they had no need of the services of a skilled nurse, instead of showing their thankfulness by renewing their subscription to the nursing funds, have declined to again pay 5s. for a ticket of which they had been unable to make use. At a recent meeting it was strongly urged that every effort should be made to keep the nurse, and as a result 20 more tickets were taken in the room. It was also proposed to either get up a concert or make a house to house collection in order to secure a sum sufficient to justify the committee in retaining the services of the nurse, which were a great boon in the past year. Over 2,000 visits have been paid in 1901.

### CO-OPERATION AT NORWICH.

We are glad to learn that the Norwich Nurses' Co-operation, which was started seven years ago, is appreciated not only in the city of Norwich and in Norfolk, but also in the neighbouring counties. It is worked on the same principles as the London Co-operation, and is managed by a committee of ladies, six of whom are on the nursing staff, with a president, two vice-presidents, a secretary, and treasurer. No members are admitted who have not a certificate of at least three years' hospital training and some experience in private nursing. The address of the co-operation is 76 Prince of Wales Road, Norwich.

### DISCORD AT NEWTON ABBOT.

The inquiry by a special committee of Newton Abbot Board of Guardians into the reasons why so many assistant nurses have left the Board's service since May 1898, has been concluded. The committee decided that there was no ground for complaint against the superintendent nurse. In the discussion which followed, Dr. Ley condemned the visiting committee, as at present constituted, as a perfect sham and a farce. Mr. Sharland, the chair man of the visiting committee, complained of Dr. Ley calling for information at the Board before mentioning it to the committee. Certainly the inquiry has been fruitless in so far as the Board are now no nearer a solution of the mystery of the nurses leaving than they were before, and it has been suggested that they would do well to keep a standing advertisement in the papers. The latest development at Newton Abbot is that the visiting committee are about to consider the question of employing probationers.

### SHORT ITEMS.

The marriage of Miss Minnie Jane Wreford to Mr. Edward A. Cook, at Singapore, is announced. Sister Wreford was trained for three years at the General Hospital, Birmingham. She shortly afterwards joined the Colonial Nursing Association, and was appointed to the post of sister at the Government Hospital, Singapore, where she has worked for 18 months. She was given away by her brother, at whose house a reception was afterwards held.—A three days' bazaar was held at the Queen's Hall, Langham Place, on Wednesday, Thursday, and Friday last week, in aid of the "Sisters of the People" attached to the institution known as the "West London Mission." The bazaar was opened by the Lord Mayor and Lady Mayoress, the Countess of Stamford, and Lady Henry Somerset on the there days respectively. Among other works carried on by the sisterhood is district nursing in Marylebone and Soho. There are four nurses, trained and certificated, and working under medical supervision. The nurses live at Katharine House, Fitzroy Square.—A very successful concert and dramatic entertainment was given on the afternoon of December 16th at the Grand Stand, Ascot, for the object of raising a fund for the erection and equipment of an operating theatre at the Royal Victoria Nursing Home at Ascot. The committee hope, after paying all expenses, to have in hand the substantial sum of £500.—The *Bavarian* left Capetown for England on December 17th with Nursing Sisters E. M. Beck and M. E. Howell on board as invalids, and Nursing Sisters M. H. Bruce, M. Whiteman, G. Napier, M. Rorke, and A. Kuys returning home.

### Lectures to Nurses on Anatomy.

By W. Johnson Smith, F.R.C.S., Principal Medical Officer, Seamen's Hospital, Greenwich.

LECTURE VII.—THE SKULL (*continued*).

*The lower surface of the skull*, with its irregular projections of bone and its numerous holes (foramina) of varying size, presents a strong contrast to the smooth and unbroken surface of the vault. In front we see the upper row of teeth and the *hard palate* forming the roof of the mouth. Behind the palate are two large orifices separated by a thin plate of bone. These are the *posterior openings of the nose.* We come now to a large bone occupying a considerable portion of the base and extending to the temples. This bone, the outlines of which can be readily traced, bears some resemblance to a bat with outspread limbs and wings. It is called the *sphenoid.* Joining the central mass or the body of the sphenoid and, in the adult skull, firmly welded with it is the lower portion of the occipital bone which, as has already been stated, takes up more than half of the base of the skull. The largest of the numerous orifices presented on this surface, if we exclude the openings into the nose, is the oval opening in the basal portion of the occipital bone. This, which is called the *foramen magnum* transmits together with other structures of minor importance the upper part of the spinal cord.

Fig. 15.—Lower surface of skull.

The anterior or facial aspect of the skull presents three large openings, those of the eye-sockets or *orbital cavities*, and a central heart-shaped one forming the entrance to the nose and called the *opening of the anterior nares.* Above this opening are the two small nasal bones which give prominence to the bridge of the nose, and on either side forming a greater part of the proper skeleton of the face are two large irregularly-shaped bones which carry along their lower margins the upper row of teeth. These, which are the upper jaw bones or *maxillæ*, though apparently strong and massive, will, if they can be detached and examined apart from the rest of the skull, be found for the most part to be mere shells of thin bone enclosing each a large air-cavity called the *antrum*, which cavity sometimes gives trouble by being distended by mucous or purulent fluid. Beyond the superior maxilla on both sides is the *malar* or "cheek bone," which is so much in evidence in Mongolians and other "high-cheeked" subjects.

The face is completed below by the lower jaw or *mandible* which in its anterior horseshoe-shaped portion carries the lower row of teeth. The vertical portions of the jaw behind the teeth terminate above in two distinct processes of bone—the posterior one for articulation with the base of the skull, and the anterior one, the *coronoid process*, for the attachment of the large temporal muscle, the action of which is to bring the upper and lower jaws together—to close the jaw. The point of junction between the lower margin of the transverse part of the bone and the posterior margin of the ascending part is called the angle of the lower jaw or the *gonion.*

Fig. 16.—Front view of skull.

There remains but one pair of bones to complete the cranium. These are the temporal bones interposed on either side of the head, between the lower margin of the parietals and the base of the skull. This is in many respects an interesting bone. Among its many distinctive features are:—The thin plate (*the squamosal*), the thinnest part of the cranial wall, which fills in the side of the skull the large nipple-like process behind the oval opening of the ear, or *external auditory meatus*, which process of bone, called the *mastoid*, is honey-combed by large air cells, including one situated just behind the meatus, which, as its name *mastoid antrum* may suggest, has considerable surgical importance; a prismatic process of very hard bone, termed the *petrosal*, which forms part of the base

Fig. 17.—Side view of skull.

of the skull and contains the complex organs of hearing. Between the temporal and the malar or cheek bone passes an arch of bone—the *zygoma*—which gives attachment to one of the muscles closing the jaw, and which is very strong and prominent in carniverous animals.

Before putting our skull on one side we should take a parting glance at its interior. The inner surface of the upper detached portion or skull-cap is not so smooth, we shall find, as the outer surface, as it is marked by broad and shallow depressions resembling those formed by slight finger pressure on a lump of dough or putty, and also by fine arborescent lines; the former being the impressions made by the convoluted surface of the brain, the latter marking the channels of blood vessels. In many skulls the bone along the course of the sagittal suture resembles a piece of worm-eaten wood presenting numerous minute holes caused by the pressure of small and soft glands called *Pacchionian bodies*.

The interior of the base of the skull in which we recognise at once the *foramen magnum* presents, when viewed from the front to the back of the head, three well-marked divisions, an anterior one with a level floor corresponding to the roofs of the eye-sockets, a middle one constituted by a deep depression on either side, and posteriorly a still deeper depression corresponding to the lower part of the occipital bone. These three divisions, taken in the above order, are the *anterior*, *middle*, and *posterior fossæ* of the base of the skull.

### The Nurses of the Cancer Hospital.

A CHAT WITH THE MATRON (BY OUR COMMISSIONER).

The treatment of cancer never excited more attention than it does at the present time, and the conditions on which the nursing is carried on at the well-known Cancer Hospital in Fulham Road will be read with general interest. On the occasion of my visit the matron, Miss Rogers, afforded me every facility for seeing the nurses at their work, and of observing the remarkable contrast in the appearance of the patients in the chronic wards and those who are suffering from the disease in its earlier stages. I had also the opportunity of inspecting some of the rooms provided for the nursing staff, of noting the admirable arrangements for out-patients, the spotless kitchen, the spacious store-room, and last, but not least, the charming little chapel in which the chaplain of the hospital conducts service twice every Sunday and once in the week.

### The Course of Training.

In our subsequent chat, the question of training was first discussed, and the matron said :—

"Our period is two years, which includes two months' trial. If at the end of two months the candidate is accepted as a probationer, she has given to her three caps, six aprons, and three dresses."

"Do your probationers stay on as assistant nurses?"

"Sometimes. In any case, if at the end of the two years they pass the examination, they have a certificate given to them by the committee, signed by the Chairman of the Board, the house surgeon, and myself."

"What salary is given during the time of probation?"

"The first year £8, and the second £12. Probationers who remain as assistant nurses receive from £18 to £20 The age of admission is from 21 to 28. It is a great advantage to many to be able to enter early, particularly as if a nurse passes our examination she is always, if she desires, taken at once as a probationer at a general hospital."

"The general hospitals must be glad to get them?"

"I think that they are. At any rate, our nurses usually go from here to general hospitals or the best infirmaries, and some of the matrons apply to me for probationers. While our probationers are serving their time here they frequently discharge the duties of assistant nurses."

"Do you have plenty of suitable applications for probationerships?"

"We have no difficulty in filling up vacancies, and I have an ample number from which to choose. But I occasionally wish that I could obtain probationers with the knowledge which ours possess when they enter other hospitals."

"Are the usual lectures given?"

"Yes; and in addition to lectures on anatomy and physiology we have lately introduced lectures on elementary nursing, which are given by the sister in charge of the theatre, who explains the use of the instruments. The committee award three prizes at the examinations to those who answer best."

"How much off-duty time do you allow?"

"Two hours every day, one half-day weekly, and half a day every other Sunday. The probationers have a fortnight's holiday each year."

"Is the duty varied?"

"Yes; they start with six months on day duty, followed by three months on night duty. Then they are changed as often as possible so as to take duty in different wards day and night. They are also taken periodically to the theatre. Two probationers are always there. Though the sister is in charge, a staff nurse goes in with the patient."

"How many nurses are on duty during the night?"

"Seven, and the night superintendent, who is responsible for the probationers, and sees that they get up and go to bed at the proper time. The day probationers are, of course, under the charge of the ward sisters."

"What is the number of wards?"

"Nine. One has just been added to the large ward on the top floor."

"And the number of sisters?"

"Seven with the theatre sister, or eight counting the night superintendent. Three of the wards are small. The sisters also have charge of two small wards and have three probationers to assist them. The largest ward has 14 beds and the smallest four. There are two assistant nurses and 21 probationers.. Since we took in chronic cases we have had to considerably increase the staff. There is an isolated cottage for cases of blood poisoning and erysipelas, but for these we have an outside nurse."

"When do the probationers on day duty go into the wards?"

"At 7.30. They breakfast at 7, dine at 12, have tea from 4.30 to 5.30, and supper at 8.30. The night nurses rise at 7.30 P.M., have breakfast at 8.30 P.M., and dinner at 10 A.M.

### Diet of Night Nurses.

"There has been so much discussion lately about the diet of night nurses that I should be glad if you would kindly give me details of the food you give your nurses on night duty."

"For breakfast they can have hot bacon, cold ham, fish, eggs, sardines, sausages, tongue, or bloater paste, jam and marmalade. The variety for dinner includes roast and boiled joints, haricots, Irish stew, rabbits, meat pies and puddings, as well as sweets of all kinds, or milk puddings and fruit. In the night the nurses can have eggs, sausages, tea, cocoa, and any quantity of milk they like. Certainly, there is no stint as to diet, and our night nurses fare just as well as the day nurses."

"When do night nurses go to bed?"

"Generally between one and two in the afternoon. They are called up between 7.30 and 8, and go off duty at 8 A.M. Once every month they have a night off, and can sleep out if they choose. I keep them on night duty for three months, for I confess I do not agree with the more constant changes which are made in some institutions."

"What are the hours of the sisters?"

"The sisters, or head nurses as we more frequently call them, are on duty at 8 A.M. They breakfast at 8.30, dine from 1 to 2, and have tea and supper at the same time as the probationers. They are off duty two hours daily, a day and a night once a month, and half a day alternate Sundays. Each of them has a month's holiday in the year. With regard to rooms, each head nurse has a bed-sitting-room to herself."

"Adjoining the ward, as I saw just now?"

"Yes; all but the night superintendent, who resides in the Nurses' Home, which was specially built for the night nurses, and opened in the early part of the present year. Each night nurse has a separate bedroom."

"But not the day nurses?"

"As far as posible; but some of the day nurses share a room."

"The salary of the head nurses," continued the matron, "commences at £30 and rises to £40. Uniform is also provided."

"Is the wearing of it obligatory?"

"Only indoors. In fact, outdoor uniform is not provided. The head nurses are given blue merino dresses, with linen aprons, collars, and caps. The probationers wear washing print dresses and caps."

"Do the sisters stay for a long time?"

"One has been here for 14 years, and others for some time. Of course, the head nurses have all had training at a general hospital."

"You have been matron for a good many years?"

"I came as assistant matron, 17 years ago, and in the second year I was appointed matron. The hospital has been very much improved since then, and operative cases have increased wonderfully. They still continue to increase."

"Is there any scheme of pension in connection with the hospital?"

"No; but annuities are granted by the authorities to old nurses."

### The Work.

"Do you consider the work trying?"

"Undoubtedly it is, especially in the chronic wards. On the other hand, the operations are most interesting, and the experience is very valuable. Altogether, I think the strain is not so great as in a general hospital."

"Is there any religious test in force?"

"None whatever. Attendance at the Chapel services is not compulsory. But I never find that a Nonconformist nurse declines to attend. The chaplain visits the patients every day. They enjoy the services. As you are aware, the Bishop of London last month unveiled the window placed in the chapel by the Cordwainer's Company to the memory of the founder. He was very pleased with the hospital, and he has had personal experience of the care bestowed by the nurses on the patients."

"When was that?"

"At the time he was head of the Oxford House, he came here with a patient and sat up all night with him."

"Lastly, as well as free admission for all suffering from cancer, you have free entertainments for them?"

"Yes, they are rather a feature, and the nurses always do their share to render them successful. Dramatic entertainments and concerts are given in the winter, and garden parties in the summer, the latter at the expense of a lady who takes a great interest in the hospital. The nurses attend in turns. At Christmas they give themselves up entirely to the amusement of the patients. Their own recreations are tennis and croquet, and there is an excellent library from which they can have fresh books as often as they please, without any kind of payment."

### Appointments.

Ballymena Union Infirmary.—Miss Elizabeth Stack has been appointed superintendent nurse. She was trained at Belfast Union Infirmary and Fern Hospital, and has since been nurse at the Infirmary and at the Samaritan Hospital, Belfast.

Brook Fever Hospital, Shooter's Hill.—Miss Margaret McLellan has been appointed housekeeper. She was trained at Perth Royal Infirmary, and has since been charge nurse and night superintendent at Brook Fever Hospital.

Chertsey Union Infirmary.—Miss Minnie Eliza Hopwood has been appointed staff night nurse She was trained at the City of London Infirmary, Bow Road, E., and has since been nurse at the Western Hospital, Segrave Road, Fulham.

Fylde District Hospital, near Lytham. — Miss Frances C. MacCartis has been appointed matron. She was trained for three and a-half years at the Mater Misericordiæ Hospital, Dublin. She has since been lady superintendent Mercers' Hospital, Cork, Queen's nurse in Dublin and Bolton, and for the last year in charge of the Enteric Hospital, Bolton.

Islington Infirmary.—Miss Harriet Orrell and Miss Margaret Hughes have been appointed sisters. Miss Orrell was trained at East Dulwich Infirmary, and has since been staff nurse at Islington Infirmary. Miss Hughes was trained at Mill Road Infirmary, Liverpool, where she has since for two years been theatre sister.

Kingston-on-Thames Infirmary.—Miss M. van Raalte has been appointed night sister, and Miss K. H. Todd and Miss H. M. Garwood ward sisters. Mrs. van Raalte was trained for three years at the Sussex County Hospital and the City of London Lying-in Hospital. At the latter she was afterwards night sister, and she holds the L O.S. certificate. Miss Todd was trained for three years at the Royal Infirmary, Bristol, and has since been charge nurse at the Park Hospital, London; sister at Rushill Hospital, Glasgow; and charge nurse at the North-Eastern Hospital, Tottenham. Miss Garwood was trained for one year at the London Hospital and for two at the National Hospital for the Paralysed, Queen Square, London. She has since been staff nurse at Windsor and Eton Royal Infirmary, sister-in-charge of Dartford Cottage Hospital, and head nurse at the Medical and Surgical Home, New Cavendish Street, London.

Lancaster Corporation Sanatorium.—Miss Mildred Lanyon has been appointed matron. She was trained at the Homœpathic Hospital, London, and has since been matron at the Isolation Hospital, Kettering.

Poor Law Hospital, Salterhebble, Halifax.—Miss Florence Frost has been appointed matron and Miss Eva Ward assistant matron. Miss Frost was trained at the Royal Infirmary, Wigan, and has since been charge nurse at St. Luke's Hospital, Halifax, sister at Leeds Union Infirmary, and senior sister and assistant matron at the Metropolitan Convalescent Institution, Walton-on-Thames. She has also been in charge of the Riviera Nursing Institution, Nice. Miss Ward was trained at Salford Infirmary, and has since been on the staff of the Hope Hospital, Pendleton, Manchester.

Stockport Infirmary.—Miss Madeleine Wilson has been appointed sister of children's ward. She was trained at the Hospital for Sick Children, Great Ormond Street, London, and the City Fever Hospital, Birmingham, where she has since been staff nurse.

Stroud General Hospital.—Miss M. G. Baxter has been appointed charge nurse. She was trained at Burton-on-Trent Infirmary for three years, and has since been charge nurse at Noble's Hospital, Douglas, Isle of Man.

Victoria Cottage Hospital, Worksop.—Miss Gertrude E. Bailey has been appointed matron. She was trained at the County Hospital, Lincoln, where she subsequently held the post of sister of the male surgical and accident ward for two years. Before going to Lincoln she was at the Royal Alexandra Hospital and Convalescent Home, Rhyl, for 15 months.

### Her Christmas Boy.

TOLD BY A NURSE.

SHE sat alone, looking into the fire. She was very tired, for to be parish nurse in a widely-scattered district like Staverton, meant a great deal of tramping over the moorland, and toiling up and down steep, and often muddy lanes. All day she had been obliged to go on foot from one patient to another—the weather making a bicycle out of the question—and this evening Nurse Hester Donaldson was very tired, and glad to shut herself into her cosy room in the cottage where she lodged. Though small, the room was comfortable and homelike; the firelight played on some of her favourite pictures on the wall, the circle of lamplight fell on a table strewn with books and magazines sent by Hester's family to please her at Christmas time.

She drew her armchair close to the fire, and the table close to her chair, prepared to rest and enjoy herself.

Outside, the wind moaned dismally, a scutter of rain fell against the window pane. Inside, she could hear the voices of her landlady and the children coming cheerfully from the kitchen across the passage.

"How glad I am I finished my rounds before the wind and rain began," she thought, as she lay back in her chair looking at the glowing fire. She saw many pictures amongst the red coals; for it was near Christmas, and Christmas awakens memories that sleep the rest of the year. Home, and friends and glad days of youth and joy came springing before Hester's eyes as the flames leapt in the grate, but one picture came oftener, and tarried longer than the others, and watching it, the smile died from her lips.

The background of the picture was a garden in spring time —daffodils gleamed against the brown earth; the sky showed pale and blue through the boughs of an almond tree pink with blossom. A man stood beside her on the daisy-covered lawn, and his face looked at her out of the fire with loving steadfast eyes.

"Only one more voyage, sweetheart, and then we will be married." Her sailor lover's hands had grasped her's closely; his kiss lingered on her lips; and then he had gone away across the daisies, and under the almond tree, where a blackbird whispered of spring and love.

"Gone away" for his last voyage from which he had never come back!

No one ever knew the fate of the ship in which he sailed; but she never came into port; and through the years that followed Hester had learnt the meaning of heartache and of hope deferred that ends at last in an anguish of despair, learning as well to take hold of her life and live it bravely for others, now that her own happiness was dead.

She sat up presently, drank her tea, and buried herself in her magazines, congratulating herself afresh on having finished her work for the day.

Suddenly there was a knock on her door.

"If you please, nurse," said her landlady, "there's a boy wants you to go to Plover's Hollow."

Hester's heart sank. She turned to question the messenger, who stood just behind the landlady. "Was it urgent?" she asked. And the boy replied that the sick man was a stranger lodging with his mother for the night; that he seemed "powerful queer," and please would nurse come. "The man says he's going away to-morrow," the boy added. "Mother says he's too ill to tramp on, but he says he will go."

Hester looked round her cosy room, realising how dreadfully tired she was. The wind had risen, the rain was dashing in torrents against the window.

"Don't 'e go, Nurse," said the landlady, "'tis a terrible night; sure the morning will do well enough, and 'tis only for a tramp after all—and Plover's Hollow a mile and more away."

Oh, yes! Hester had remembered that fact, and the weary walk that getting there involved; and certainly it was a terrible night, and if the man said he was fit to start again to-morrow, surely it would do perfectly well if she went to see him the first thing in the morning!

A fresh gust shook the house. Hester turned to the messenger.

"Tell your mother I will come over in the——"

Her sentence broke off suddenly. Some words spoken long ago by Stephen Rand, the man she loved, flashed into her mind—"One who never turned his back, but marched breast forward." Should she turn her back now upon a plain and obvious duty?

Her sentence remained unfinished. She took her cloak from the peg, put on her bonnet—all without a word—then turned to the boy with a smile.

"I am ready now," she said quietly, and unheeding the remonstrances of the good woman of the house, she stepped out into the darkness and storm. To her dying day she will never forget that walk. The wind tore across the open moor, shrieking and wailing like the spirits of the lost, and buffetting her until she could hardly stand against it. The rain came down with such force that long before she reached her destination she was almost drenched to the skin The climb up the steep hillside seemed interminable, the long descent into the hollow was like a nightmare. It was pitch black, a torrent of water raced down the stony lane, and she and the boy slipped and stumbled countless times as they picked their way through the darkness. By the time they reached Plover's Hollow she felt utterly worn out and weary beyond words. She was dimly conscious that her tired limbs would not have carried her an inch further!

The woman who admitted her looked at her with eyes full of compunction, "Well there, nurse," she said, "I didn't ought to have brought you out on such a night, but I was that nervous. The poor fellow seems better now, he says he'll go on to-morrow, happen what may."

"Where is he?" Hester asked faintly, "I had better see him. Taking off her wet cloak she followed the woman into the little front room of the cottage where upon a couch lay a man, his face turned to the wall, his hand hanging down limply

Hester crossed to his side.

"Can I do anything for you?" she began, "I am a nurse, and ——"

Before she could say more the man turned quickly, with a low exclamation.

"Hester! why, Hester, is it you?"

She stepped back a pace, her eyes wide and startled.

"Stephen!" she faltered, "I thought —— "

But again he did not give her time to speak. He rose and staggered weakly across to her with tottering steps. He looked like a ghost of his old self, so worn and haggard was his face, his hair so grey, but the eyes that looked into Hester's were the steadfast eyes she remembered, the voice the tender voice of her young lover.

He drew her hands into his close clasp; he drew her into his arms and kissed her, and some of the bewilderment died out of her face.

"Is it really you?" she gasped.

"Really I! Did you think I was a ghost? No wonder you could not believe I was flesh and blood, after all these years. I was going on to-morrow to try and find you in the old place."

"Then we might never have met," she cried, clinging to him, "for my people have moved, and the old home is broken up. We might never have met, if I had not come out to see you to-night!"

It was a strange story he had to tell her, when he sat in her little room next day; a story of shipwreck, of long weary years in an island in the Southern sea, and of his tramp towards London, hindered by the attack of malaria that had driven him to the cottage in Plover's Hollow.

"But it is all right now," he ended simply.

"Yes, all right now, but so nearly all wrong," she answered. "I nearly did not go out last night, because of the storm, and because I was tired and selfish. I almost stayed at home. I tried to shirk my duty, and if I had done it I should have lost——" She paused for very excess of feeling.

"You would have lost your Christmas box," he said gaily, "and a big substantial one too. You see I have come just now especially to give it you—myself!"

### Christmas in the Holy Land.

By a Nurse.

Christmas was rapidly approaching, and we nurses, with hearts turned towards the Dear Homeland, consulted together to think how we could best celebrate the festival in our little hospital on Mount Carmel. About two days before Christmas Day we collected as many evergreens as we could, and decorated the wards, the hall, and our dining-room.

Those patients who were Christians shared our enthusiasm, but the Jews and Moslems did not take much interest in the preparations. One patient especially—a young Englishman—sympathised with us and wished he could have helped us. That, however, was impossible, as he was only lately convalescent after typhoid, and not fit to exert himself.

On Christmas Eve the English mail arrived, bringing for us loving greetings from across the seas. At midnight a fellow-nurse and myself went to the Marionite Church, where an impressive service was held. The church was crowded, but our faithful servant, Selim, managed to secure seats for us in a good position for observation—and what a curious congregation it was.

All the women, most of them robed in white, sat together at the back; and the men, wearing their fezs, in the front part of the church. The altar was brilliantly lighted, and the numerous artificial flowers showed their stiff, glary faces with unusual conspicuousness. On the north side of the altar was a cradle, and in the cradle a representation of the Holy Infant. Much of the service seemed to consist of a kind of anthem sung round the cradle. It was altogether peculiar, but most interesting. We did not stay till quite the end, as we knew what a short night's rest was in store for us. We went to bed at about a quarter to one, and at 6 A.M. I awoke to realise that I was actually in the land of our Saviour's birth on His Birthday. I looked towards the Gallilean hills, over which the sun would later on rise, and felt an overwhelming burst of gratitude that I had been permitted to be there—in Gallilee—on Christmas Day.

The only thing to damp my ardour was a little cloud on the horizon, which, as the morning wore on, joined many others and then came down in a slow, continuous rain.

After an early service in the little English church we were greeted with a carol sung by some English-speaking native girls. The one incident I remember about it was that they brought in each of our names very frequently, so I suppose it was "a hymn of their own composing."

All the morning the loud front-door bell of the hospital was sounded repeatedly.

Many of our neighbouring friends sent us presents, and it is etiquette in the East to send a gift to your friend by direct messenger; so the servants of our many kind friends and acquaintances were very busy that morning. We had a real English dinner, and gave the hospital servants one of our plum-puddings, with which they were hugely delighted and amused.

Later in the day we played the harmonium and sang to the patients.

I think what you miss abroad as much as anything on Christmas Day, is an English fire! We did not need fires, but had we wanted them the nearest approach to a fire in Syria is a native fireplace which you move about the room as you like, and which, of course, burns charcoal. On Christmas Day we had no really bad cases in the hospital, but a few days afterwards an old German woman became very ill indeed.

One day, when we were giving a Christmas tree and tea to all the English-speaking children—natives or otherwise—our attention was very much divided between the sick woman downstairs and the children above. But as we were three nurses with only one severe case in the hospital, one of us was always with the poor woman, and she was too far away to hear the gaiety in another part of the building. After the children had gone home, and quiet reigned everywhere, and only the dying woman and myself—the night nurse—were awake, I thought of the angels' song at Bethlehem—a distance of only two days' journey from our hospital—nearly 2,000 years ago. And throughout that dreary vigil I did not seem alone; and Eleanor Hamilton King's beautiful lines—

He gives His angels charge of those who sleep,
But He Himself watches with those who wake, came to my mind; His presence seemed very real as I sat by the bedside of the dying German woman.

Not long afterwards He took her away. Her last Christmas had been spent in the land of His birth; her New Year of eternity began in the Great Beyond.

### Three Magazines.

The *Boy's Own Paper* for December is full of bright stories and contains several capital articles on Sport. The frontispiece alone will ensure the success of the number, being a large coloured plate of all the British war medals and decorations. Readers of the December number of the *Sunday at Home* will be interested in articles written by the Rev. Hubert Brooke and the Rev. C. Silvester Horne. To the *Leisure Hour* for December Mrs. Belloc Lowndes has contributed an illustrated article on "Journalism as a Profession for Women," Mr. W. J. Gordon writes on "Toys and Toyland," and Mrs. Mayne Reid tells of an adventurous excursion in America with her late husband, Captain Mayne Reid.

### To Nurses.

We invite contributions from any of our readers, and shall be glad to pay for "Notes on News from the Nursing World," or for articles describing nursing experiences, or dealing with any nursing question from an original point of view. The minimum payment for contributions is 5s., but we welcome interesting contributions of a column, or a page, in length. It may be added that notices of appointments, entertainments, presentations, and deaths are not paid for, but that we are always glad to receive them. All rejected manuscripts are returned in due course, and all payments for manuscripts used are made as early as possible after the beginning of each quarter.

### Everybody's Opinion.

[Correspondence on all subjects is invited, but we cannot in any way be responsible for the opinions expressed by our correspondents. No communication can be entertained if the name and address of the correspondent are not given as a guarantee of good faith, but not necessarily for publication. All correspondents should write on one side of the paper only.]

### THE NURSES OF ST. OLAVE'S INFIRMARY.

"The Matron" writes: There is just one little mistake in your correspondent's report, which is probably due to a misunderstanding of words. The nurses left—they were not dismissed. I believe we have not had to do more than accept a resignation since we began to train our own probationers, and it is rare for a nurse to leave except at the expiration of her training. Many of our probationers become our charge nurses in due time. The nurses who left had all just completed their training, and there was no quarrel with the institution.

### A QUESTION OF AUTHORITY.

"D." writes: I am on the staff of a small cottage hospital and am, *ex officio*, a member of the house committee. This committee is anxious to obtain the services of a trained nurse in addition to the matron. The lay members are of opinion that applications for the post should be addressed to the secretary or treasurer and be dealt with by the committee. The medical members are of opinion that the selection should rest entirely with the matron, and that she should deal with all applications and interview in the first instance all candidates for the post, and when she has found one whom she considers suitable present her to the committee for election. I have always been under the impression that the selection of nurses was a matter left entirely to the matron, and that she was supreme in the nursing department, being, of course, responsible to the committee for the proper nursing of the patients committed to her care, and also for the training of probationers. I shall esteem it a great favour if you will kindly give me the benefit of your views and experience in an early issue of The Hospital.

[It is essential for the good management of the hospital that the matron should have complete authority over the nursing staff, and this is impossible unless she has the power of both appointing and discharging.—Editor The Hospital.]

### THE NURSE'S NEVER.

"Vigilant" writes: I am not a hospital nurse, but I have seen a great deal of sickness and nursing, and had a pretty large experience of nurses. The letter of "A Royal Alexandra Nurse" is, I consider, the very essence of common sense, and I have no hesitation in saying that a fully-qualified hospital nurse no more needs reminding of her duties than a fully-qualified physician or surgeon needs to be told how to treat his various cases. Being human, everyone is liable, now and again, to make a mistake. But, always supposing that a nurse has, firstly, an inherent love of nursing, and secondly, a capacity for nursing, a "thoroughly-trained" nurse is so fitted by her training for her duties, that she will know instinctively what to do, and what to avoid doing. I do not agree with "Matron" in her remark that "the best nurses sometimes need reminding of their duties," and I think that, besides showing a want of sympathy between "Matron" and her nurses, such a state of affairs reflects discreditably on the institution, and on the training of the nurses concerned.

### NURSES AND THEIR FOOD.

"A Sister" writes: I write to tell you what immense amusement your "Everybody's Opinion" column affords our household, *i.e.*, regarding the "Nurse's Never." Every head of an institution knows how extremely valuable and rare the nurses must be who live up to the standard of the "Nurse's Never." The agonies about the nurses' food is amazing! Why is it that shop assistants, dressmakers, milliners, artists, typists, teachers of all kinds, and other women who work for their living do not continually wail about their food and comforts? Usually it is nurses and servants who do so. Again, nurses are chiefly drawn from the above-mentioned workers. Many gladly leave their proper work to enter the nursing "profession." I have observed that, as a rule, nurses look the best fed and cared for of any women, and personally I consider that the hospital chiefs are to be congratulated on their generally comfortable well-fed condition and appearance. It is possible that if the latter were more content and less expensive in their requirements people would appreciate them more, and probably marriages would be more frequent. It would require a really good income, better than many men can command, to maintain the average working-woman nurse. The "working-gentlewoman nurse" does not require so much maintenance, and does not grumble. I may state that very many probationers, standing on their working-market value, would simply die for want of the plain, good, and abundant food supplied them in hospitals.

### DANGERS OF THE TELEPHONE.

"A Hospital Nurse" writes: In the interesting article in The Hospital directing attention to the possible drawbacks arising from the frequent use of the telephone which is likely in the future to be as much a part of the house as the electric light and the electric bells, there is one serious omission: no mention is made of the telephone as a most dangerous source of infection for diseases of various kinds. At a time when so much thought and attention are devoted to the study of consumption it seems curious that no one has pointed out the dangers of the telephone as a transmitter of germs. In a first-class hotel where I am now staying with a patient the public telephone is in a simply filthy condition, and has to be thoroughly cleaned before using, a large quantity of grease and dirt being left in the cloth. If such is the state of affairs in a good hotel, one's imagination fails to realise what the telephone must be like in a public call office, where all sorts and conditions of men congregate. In the health resorts many invalids use the telephone and add a still greater danger to this instrument. In the interest of the public it is most necessary that some remedy should be found if diseases are not to be scattered broadcast over the land. I would suggest that by the side of every receiver there should be placed a saucer containing a solution of perchloride of mercury or carbolic, and a sponger for wiping the receiver immediately after it is used, so that all germs may be killed at once.

### THE "ENGAGED" PROBATIONER.

"E. M." writes: I do not think it is necessary for matrons to refuse to accept a probationer because she happens to be engaged to be married. "There is many a slip between cup and lip," and if it is a case of waiting for years, the waiting cannot be better spent than under the discipline of a good training school; for whatever position in life the nurse may be going to enter after, it will be more than useful to her. On the other hand, I must confess that, as a rule, probationers who are engaged do not make the best nurses. Their thoughts and attention are often distracted by outside influences and they are generally too ready to get off duty at their stated time, and perhaps in consequence hurry over a patient or leave some work undone for the sake of an extra few minutes out of the ward. I do not think my late matron would have taken any nurse had she known she was betrothed; in fact, if we were too eager to get our letters from friends, which we saw were for us in the locked letter-box, she purposely kept them back, and then reminded us that probationers without friends were more likely to get to the top of the tree. Looking back upon those years, I thank her for the hard discipline, although at the time I regarded it as almost inhuman. After all, we are only ordinary women, with women's hearts, so I think the question should not be asked when engaging a nurse; but she should be allowed to take her chance with the others on her trial, and kept for the merits of her work and intelligence alone.

### Echoes from the Outside World.

Alike in the Outside World and within the walls of the hospitals the same words are being echoed on every side, "A Happy Christmas." Most sincerely do I repeat the good wish to all my readers, mingled with many hopes that satisfaction and pleasure and gratitude on the part of the patients may do something to ease the tired feet and the tired heads of many nurses about this time. As to the matron who has to cater for the wants of her household this Christmas time she will probably have discovered ere now that her bills have reached a higher figure than they have touched for some time. Only one item, and that a very important part of the fare, is particularly reasonable. Turkeys are good and cheap. British birds are, of course, charged at a higher rate than that asked for foreigners, but the ample supply of the latter enables those who would otherwise be obliged to content themselves with beef to indulge in a bird. This is a matter for congratulation, as beef has risen in price, and some butchers are actually asking 13d. a pound for the best sirloins, and then maintain that even then there is little profit to be got out of the transaction. Suet is even higher in proportion. The usual cost is 8d. a pound; 10d. and 1s. is now being demanded and paid. Those who left their pudding-making till late will have been angry at the extra expense; but if it makes them more inclined to get forward another year, and avoid a rush at the end, it may be an experience worth having. Game is cheap, and mistletoe more reasonable than it has been for ages, but berried holly is at a fabulous figure, and even a bit for the top of the pudding costs a couple of pence. Christmas trees are to be had for next door to nothing, and good oranges, for their decoration first and for consumption afterwards, are 40 for a shilling.

Although Signor Marconi has been so busy making experiments with his wireless telegraphy at St. John's, Newfoundland, he has managed to find time for the necessary preparations for an experiment of another kind. It is reported that early in the new year he will be married to Miss Josephine Holman, of Indianapolis. Such an announcement is naturally of interest, because the name of the young inventor is now world-renowned; and if his last success fulfils his expectations the honour accorded to him will, of course, be increased a thousandfold. Ever since the first time of signalling by means of air-waves, Marconi has been gradually increasing the distances over which he had made the message travel. Lately he has gone to Newfoundland to see whether it would not be possible to receive a message there from England He cabled directions to his assistants in charge of the experimental station at Poldhu, Cornwall, that every day, at intervals of five minutes between the hours of 3 and 6 P.M., a pre-arranged signal, the letter S in the Morse alphabet, expressed by three "dots," should be dispatched. To receive this signal, kites with a vertical aerial wire were sent up into the air to the necessary height, a specially sensitive telephone being attached to the receiving instrument. On the first day the kite broke, but on the next the vibration of the three dots were, it is said, distinctly felt by Marconi and his assistants. The following day the success was repeated. Then the Anglo-American Telegraph Company served Signor Marconi with a notice that if he made any more experiments on their territory they would take legal proceedings. The company have a monopoly by which they have the sole right to receive and transmit ocean telegraph messages from St. John's for 50 years. Two years of the monopoly are still unexpired. It is hoped that the Anglo-American Telegraph Company will withdraw their objection to the experiments; but, if not, Signor Marconi will have to wait a little while or experiment elsewhere.

If the "Horos" trial and the very proper sentences pronounced upon two of the most dangerous and disgusting of modern criminals has the result of opening the eyes of credulous women to the peril of playing with fire in the shape of matrimonial advertisements, the publication of the loathsome evidence will to some extent have done good. I think that there is considerable force in the contention that respectable newspapers should not allow their columns to be used for such a purpose, unless at any rate they are satisfied that *bona fide* intentions are entertained; and, even then, the way to a happy married life does not lie through the medium of advertisements. Nurses are not exempt from folly, and it is possible that some whose experience of the world has been slender may have been tempted by a clever decoy to commence a correspondence with a plausible scoundrel. The only safe course is to let all matrimonial advertisements severely alone. I was much struck a day or two ago, when we were discussing the desirability that every girl should be brought up so that she could earn her own livelihood, if necessary, when she left school, by the declaration made by a woman of education and social position, that she thought "the profession of matrimony" was the best of all professions for a woman. To those of my sex who take this view, answering advertisements for a wife may be a part of their training; but the others—I trust, the vast majority—who look at matrimony in its true light, as primarily a state to be entered upon with Divine sanction rather than as a means of providing for material wants, cannot do otherwise than regard the advertising business with aversion.

Conditional promises are very satisfactory when there is a reasonable hope of fulfilling the condition. The rector of All Hallows, London Wall, who is doing his best to supplement the rest and shelter already provided for working girls in the church by a similar provision for working men in an extension of the building in the churchyard, has been promised £100 by the Goldsmiths' Company as soon as the remainder of the sum needed is subscribed. This means that £500 has yet to be obtained. I should have thought that in trying to increase the practical use of a City church the rector would have found ample support from the professed Church people who coin money within a stone's throw of London Wall.

I wonder whether most grown-up people are as fond of children's plays as I am? I suppose not, or the theatres at Christmas time would be largely occupied by grown-ups, who perhaps had no little ones to "treat." Every year I go to a child's play for pure enjoyment on my own part, quite without reference to any small people who may accompany me, and I can truthfully say that I have seldom had a better time than I did the other day at the Vaudeville Theatre. "Blue-bell in Fairyland" is delightful from every point of view. The story is a real child's story, not a collection of topical allusions, far-fetched jokes, and beautiful grouping and dancing; the music is tuneful, and the songs neither too long nor too difficult for small minds to understand; the scenery and the dances are both excellent; the fairies and mortals are all well trained, and the flower-girl, the heroine, is all that the children would like her to be if she had been made to their express order. She is pretty and poor, good and sweet, and withal is pourtrayed by Miss Ellaline Terriss. All who remember Miss Terriss as "Cinderella" at the Lyceum a few years back, will agree that "Blue-bell" is quite as charming as the lady of the glass slipper, and that is saying a good deal. Mr. Hicks, as the crossing-sweeper in one part, and the Sleepy King in another, shines in both characters, and young and old will alike be charmed with Blue-bell's little sisters, Miss Phyllis Dare and Miss Winifred Hall, with Miss Molly Moore, who is a doll, and with the beautiful little dancer, Miss Dorothy Frostick, who is Will o' the Wisp. I can only hope that those nurses who go to the Vaudeville will enjoy the entertainment as much as I did.

### A Book and its Story.

### A STORY OF MIDDLE AGE.*

The name of F. F. Montrésor on the title-page of a book is a guarantee that it is interesting, and in her latest novel, "The Alien," the authoress displays the gift of vivid characterisation, of delicate humour, and subtle insight, combined with a style which is charming and holds the reader's attention from the first page to the last, which we have learnt to expect. A story not of the Middle Ages, which at first glance the title suggested, but of the far less romantic period in lives of either sex from an emotional standpoint, viz. middle age. It was a bold experiment on the part of the authoress to add this sub-title, and to announce at once that she had left the hackneyed path of youthful sentiment, and placed her characters on a more mature plane. Wisely these characters are limited, there is no over-crowding, neither are there any gaps to fill. The incidental figures necessary to the plot are chosen with care, and each fill their place with point. But it is in the four leading characters, Esther Mordaunt, Cousin Rebecca, Major Curtis Iredale, and the Alien, Jaspar, that the vitality of the story centres.

Esther "was well into the thirties when she became involved in this story of middle age." Twenty-two years previously her father's cousin by marriage had undertaken the care of his orphaned children. Mrs. Mordaunt, Cousin Rebecca, married off the two younger sisters, Rose and Lily, in their first season in town, and then turned her attention to Esther. She invited her to Applehurst in order that she might judge of her prospects before risking a journey to town. From this it may be well seen that "Cousin Rebecca" was no sentimentalist nor was she likely to waste her benefactions. Esther, fresh from school, walked into the long drawing-room, and met Mrs. Mordaunt's sharply penetrating glance. Mrs. Mordaunt had been beautiful in her youth; but Esther saw only a fat little old lady of slightly Oriental caste of countenance, who wore a hideous front of jet-black curls. Esther smiled in after years whenever she thought of her first encounter, and of the caustic reply which greeted her protest to Cousin Becky's criticisms of her looks. "My dear, you should never waste a fib. You do not think yourself ugly by any means; on the contrary, I can very well see that you've quite a good opinion of yourself; and mind, I don't blame you, for it is a thing that helps one through life." And yet, "in spite of an unpropitious beginning, we read, in spite of two hot tempers and quick tongues, Esther was the one of the three sisters who loved the old woman, who was never afraid of, though sometimes horribly irritated by her, and who wakened at last some answering affection." Cousin Becky's matrimonial schemes for Esther were frustrated by her becoming engaged to a lover not on the selected list of suitors, an impecunious naval lieutenant, who was killed in action a few months later. So ended her days of gaiety. Cousin Rebecca could not agree with her desire to withdraw altogether from society and retire into some post where in occupation she could forget her sorrow and shake off the chain of dependence which hung at times rather heavily. "No one should renounce the world before they've met it," said Cousin Rebecca. "Here's a pretty state of things. I wish I had never taken you to London . . . and as far as I'm concerned I'd be only too glad to keep you for the rest of my life, but I'm a gouty, cranky old thing, and I can't promise you much." This all happened long before the story opens, and Esther took up her abode permanently with her shrewd, eccentric relative in the old House at Applehurst, where for generations her ancestors had lived and died. Here we find her in the gloom of a November afternoon. "The house faced north-east, and it was bitterly cold . . . it was a melancholy house with an air of austerity about it. It had no grace of architecture, but only a certain amount of grim character which redeemed it from the commonplace. . . . For twenty years the house had been her home . . . it had seen her very miserable, an Esther who secretly believed her heart was broken. She had learnt since that hearts do not break, as a rule; they only crack and are quite as serviceable as before." The Applehurst estate was entailed. Mrs. Mordaunt had inherited it from her father—"not, alas! of blessed memory." At the present moment, Major Iredale considered himself heir, Mrs. Mordaunt's only son, by her first husband, a ne'er-do-weel, having been drowned abroad twenty years previously. "Major Iredale was a widower on the wrong side of fifty; he had once come to the conclusion that Esther would look well at the head of his table. He begrudged her to Cousin Rebecca. Though no longer young, she was a very graceful woman, with a distinct charm and style of her own. The Major had been annoyed when Esther, having refused him, added, with a gleam of laughter, 'And it is very thankful you should be that I do not want to marry you, for if I did Cousin Rebecca would never forgive me." Mrs. Mordaunt resented the fact that Major Iredale was waiting for her death to take up his position of master of Applehurst. She would have preferred to have some one of nearer kin to succeed her. And he shared with her the family characteristic of an arbitrary will which clashed constantly with her own "There is no doubt, in spite of these defects, that he might have been the pride and joy of some old ladies. He was kind—when he was given way to—chivalrous to weakness, honest as daylight. He was a fine-looking man still. His dark eyes were as keen as ever . . . calculating eyes, Mrs. Mordaunt dubbed them." But the Major's keen eyes had hardly calculated for an event which was near at hand, and one that would upset his claim to an estate which he looked upon virtually as his own.

There is a curiously vivid scene in which the rival heir is described as wandering in the vicinity of the grounds of Applehurst and comes upon a blind priest holding converse with the Almighty in a ruined church at eventide. "The stranger ascended the hill briskly enough, but as he neared the tower, was arrested by the sound of a voice rising and falling in a monologue. . . . He approached softly and looked in. The ruin was unroofed, but an altar, formed of three slabs of the yellow stone of the country, was raised under what had been the east window. On the altar a silver lamp was burning and before it stood an old man, bareheaded. He threw up both his hands crying aloud. . . 'Praise be to Thee who art the Life of all living. Praise be to Thee for the joy of all Thy creatures who fly by night. Praise be to Thee for the rest of those who sleep. To Thy mercy we commend all those who suffer; to Thy infinite justice all those who sin. For ever and ever. Amen.' . . . He dropped on his knees at the Amen and covered his face with his hands—thin, nervous hands, the hands of the poet or artist." The stranger had seen many strange sights in the course of a wandering life, but this struck him as being among the most strange. "I beg your pardon, sir," said the old man, "but, now that you have spoken I know very well you are not one of my poaching parishioners; nor of this country side." "I never meant to return to England," said the stranger . . . "but something drew me across the sea. . . My real life was out there and is buried there." The stranger is "The Alien." He comes early on the scene and the reader's attention is effectually awakened with his appearance. Space does not permit further notice of this unusually fascinating book. It must be read and enjoyed for the many good points enumerated.

* "The Alien." By F. F. Montrésor. (Publishers, Methuen & Co. 1 vol. 6s.)

### For Reading to the Sick.

THE DIVINE PRESENCE.

Jesus is everywhere, is very nigh;
The Holy land is in us and around,
Grace blends with nature earth with Heaven profound;
To them of loving heart and single eye
Deep sacraments all creatures underlies.

In calm and storm, in sunshine or in shade
His presence will go with thee and give rest,
Soothing the storm and passions of the breast;
Lo! I am with you always—so He said,
Even to the end; 'tis I, Be not afraid.

*Arthur Baker.*

"That which was from the beginning." St. John speaks as if Christianity was as old as the world. And so St. Augustine and great Greek writers have loved to call it. It is as old as the world, because the light, the true light, has been for ever and everywhere shining. Only now at the end of the days, that true light has shone on its completeness—Christianity is true because it is the summing-up of all that God has to teach men in all parts of the world, and it supersedes all the more partial disclosures of the truth by comprehending and including their all in its more perfect message. And so it is a thing age-long, old as the world and new in its completeness, the message about human life that St. John was to hand on. They saw with their own eyes, as men who have not seen it with their eyes have ever since felt in their consciences, that this was truly the perfect life. If this life can be lived, then truly, in deepest, fullest sense, man is blessed in spite of all adversity; man is blessed in spite of all inequalities and disadvantages; for here, here in the living of this life; here in this fellowship with God and man; here in the human life of Jesus of Nazareth—is something altogether worth having. So they looked at it. They beheld it. They gazed upon it—so the word means. It was an actual contact with this human way of living. It was truly and verily the life of the very God, the life which lies behind and pervades all things, the life in which all things consist, that now was manifesting itself under human conditions. It was God's life; the life which is universal, the life which is absolute, the life which is final. We live by His spirit; He dwells in us, and we can dwell in Him; the Divine fellowship is for you and for me, and for everyone of us, simply if we will take the trouble to be obedient to God inwardly.—*Gore.*

Two worlds are ours: 'tis only sin
Forbids us to descry
The mystic Heaven and earth within,
Plain as the sea and sky.

Thou, Who hast given me eyes to see,
And love this sight so fair,
Give me a heart to find out Thee,
And read Thee everywhere.

A home above, a home beneath the sod,
The sun will seek the west,
The bird will seek its nest,
The heart another breast
Whereon to lean; the spirit seeks its God.

*D. Greenwell.*

### Notes and Queries.

**The Editor is always willing to answer in this column, without any fee, all reasonable questions, as soon as possible.**

**But the following rules must be carefully observed:—**

**1. Every communication must be accompanied by the name and address of the writer.**

**2. The question must always bear upon nursing, directly or indirectly.**

**If an answer is required by letter a fee of half-a-crown must be enclosed with the note containing the inquiry.**

*Probationer.*

(122) I am 17, and am anxious to begin training as a nurse, but I fear I am too young.—*M. L. M.*

You are six years too young.

I am anxious to train as a hospital nurse. Is there any institution that will take me at 19?—*J. G.*

It is possible to find an institution to take you at 19, but you are not old enough for good general training until you are 23. See list of children's hospitals in "The Nursing Profession: How and Where to Train."

I am tall, strong, healthy, and 19. Can you tell me of any hospital that would train me as a nurse?—*A. E. W.*

See reply to *J. G.* See also an account of an interview with the matron of the Cancer Hospital on page 177.

Will you kindly tell us the addresses of one or two children's hospitals where we could go as probationers?—*E. R.* and *J. R.*

See reply to *J. G.*

(1) At what ages are probationers taken at hospitals? Does any institution receive them between 18 and 19 years, and is any salary given? (2) Will my having had two hammer toes removed incapacitate me?—*D. E. W.*

See reply to *J. G.* (2) Not necessarily so.

Will you kindly tell me if Tewkesbury Hospital is a training school for nurses of good standing?—*L. S. F.*

Tewkesbury Rural Hospital is only a small institution of 20 beds, and the training would not be recognised by the Local Government Board.

*Probationer in American Hospital.*

(123) I am anxious to know if you advise me to train in an American hospital and the best way to do so.—*E. J.*

Not unless you desire to work in America. You would probably find many difficulties in the way, and it would be far wiser to train in England if you wish to work there. A list of American nurse-training schools is given in "Burdett's Hospitals and Charities."

*Assistant Matronship.*

(124) I am anxious to obtain an appointment as assistant matron in an English asylum, but I have seen no advertisement in The Hospital *Nursing Section* suitable. (1) Do you advise me to advertise in the paper; and (2) is there any other journal in which I might find what I want?—*Barantium.*

1. Yes. 2. The *Asylum News*, published at the County Asylum, Lancaster.

*Assistant Nurses.*

(125) I should be much obliged if you will kindly tell me what is the general rule with regard to the selection of assistant nurses in hospitals. Should applications be made to the matron or to the house committee? Should the selection be made by the matron or the house committee? I have always understood that the matron invariably selected her own nurses.—*Matron.*

The rule is for the matron to receive applications and to make appointments.

*Bamboo Screens.*

(126) I shall be glad if you can tell me the best place for procuring bamboo screens for use in hospital wards and the price.—*Matron.*

You can get them from any firm supplying hospital furniture.

**Standard Books of Reference.**

"The Nursing Profession: How and Where to Train." 2s. net; post free 2s. 4d.
"Burdett's Official Nursing Directory." 3s. net; post free, 3s. 4d.
"Burdett's Hospitals and Charities." 5s.
"The Nurses' Dictionary of Medical Terms." 2s.
"Burdett's Series of Nursing Text-Books." 1s. each.
"A Handbook for Nurses." (Illustrated). 5s.
"Nursing: Its Theory and Practice." New Edition. 3s. 6d.
"Helps in Sickness and to Health." Fifteenth Thousand. 5s.
"The Physiological Feeding of Infants." 1s.
"The Physiological Nursery Chart." 1s.; post free, 1s. 3d.
"Hospital Expenditure: The Commissariat." 2s. 6d.
All these are published by the Scientific Press, Ltd., and may be obtained through any bookseller or direct from the publishers, 28 and 29 Southampton Street, London, W.C.

## Figures and Tables

**Fig. 15. f1:**
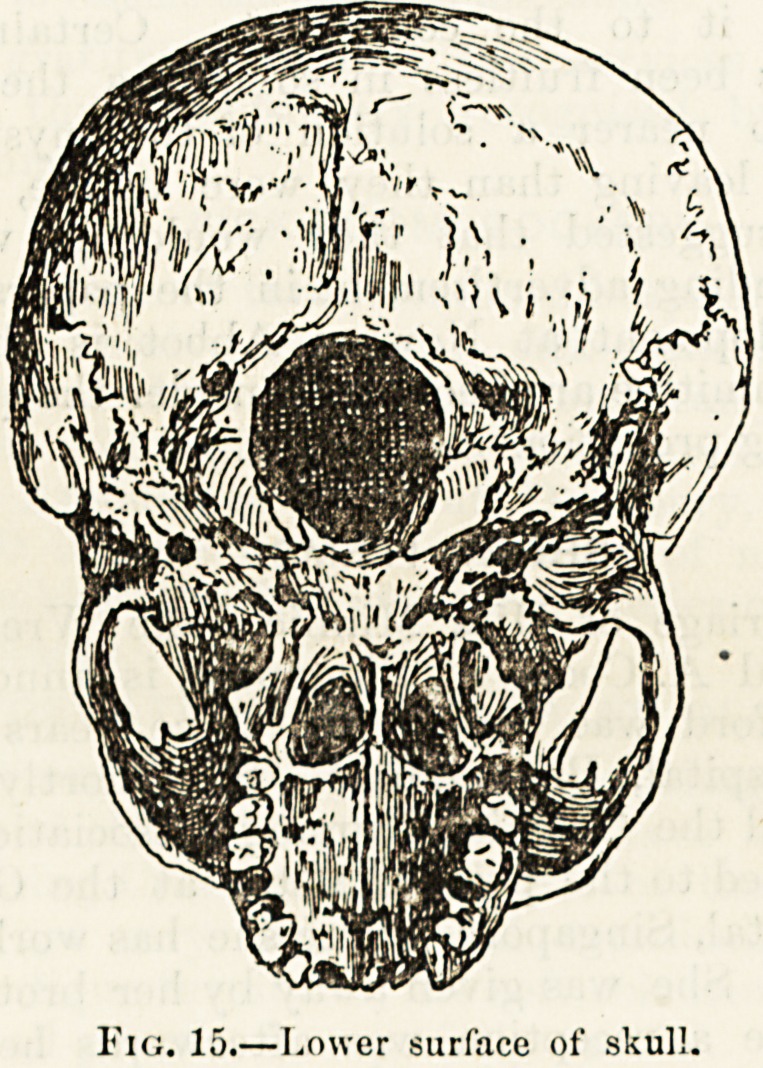


**Fig. 16. f2:**
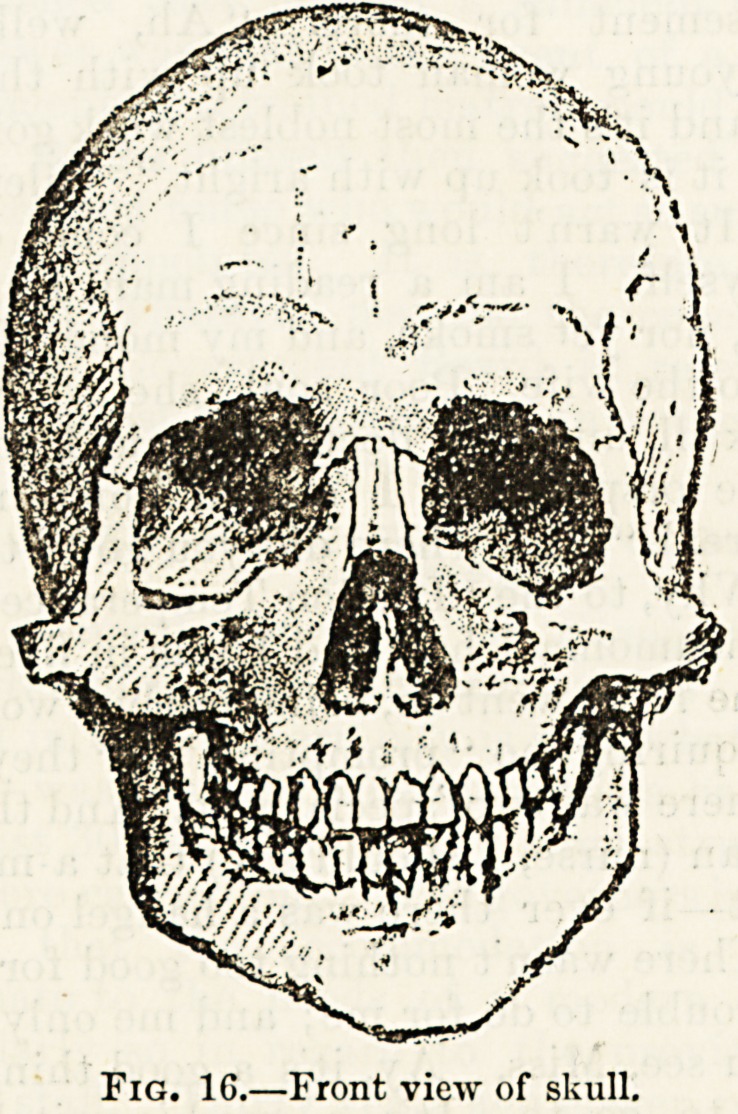


**Fig. 17. f3:**